# Quality of life during COVID-19 pandemic: a community-based study in Dakahlia governorate, Egypt

**DOI:** 10.1186/s41256-022-00246-2

**Published:** 2022-05-19

**Authors:** Shorouk Mohsen, Ragaa El-Masry, Olfat Farag Ali, Doaa Abdel-Hady

**Affiliations:** Department of Public Health and Preventive Medicine, Faculty of Medicine, Mansoura University, Mansoura, Egypt

**Keywords:** COVID-19, Quality of life, Mental health, Community-based participatory research

## Abstract

**Background:**

The sudden transmission of the novel coronavirus along with instant measures taken in response to the Coronavirus Disease 2019 **(**COVID-19) pandemic caused many new challenges adversely disturbing quality of life (QoL). The objective of this study is to measure quality of life of the public during the COVID-19 pandemic and factors affecting it among adults.

**Methods:**

This is a community-based cross-sectional household study with analytic component conducted in an agricultural area in Dakahlia governorate, Egypt and included 500 individuals. Data were collected through a structured interview, and the collected data included socio-demographic characteristics as well as some data related to their habits and comorbidities, their experience with COVID-19 and data about QoL using the COV19-Impact on Quality of Life (COV19-QoL) scale Arabic version, after assessing Content validity and reliability.

**Results:**

The total QoL score (mean ± standard deviation) is 2.3 ± 0.6 and the score for QoL in general and perception of danger on personal safety show the highest mean with 2.6 ± 0.7. The lowest mean score is related to the perception of mental health deterioration (1.9 ± 0.8). Independent predictors of the total QoL scale are sex (regression coefficient (95% CI) = 0.1 (0.02 to 0.2), p value = 0.02), monthly income (regression coefficient (95% CI) = 0.1 (0.004 to 0.2), p value = 0.04), knowing someone infected with COVID-19 (regression coefficient (95% CI) = 0.15 (0.08 to 0.3), p value = 0.001), and data collection time (regression coefficient (95% CI) = 0.1 (0.006 to 0.2), p value = 0.04).

**Conclusions:**

COVID-19 pandemic has impacted the public quality of life, particularly in terms of general quality of life and personal safety. People with substantial predictors of lower quality of life should be given more attention.

## Background

Coronavirus Disease 2019 (COVID-19) is an infectious disease of great global public health concern caused by a newly discovered SARS-CoV-2 virus [1]. COVID-19 first case in Africa was confirmed in Egypt on Feb 14, 2020 [2] and as of August 27, 2021, the COVID-19 Coronavirus pandemic website, 2021 reported a cumulative total of 287,393 confirmed COVID-19 cases with 16,706 deaths (case fatality ratio CFR:5.7%) have been reported across Egypt [3].

Like many other aspects, it is expected that the quality of life (QoL) of the public is largely affected during COVID-19 pandemic with widespread panic, anxiety and stigmatization of patients with the disease [4]. According to the World Health Organization, QoL is defined as ‘the individual’s perception of his or her position in life, within the cultural context and value system he or she lives in, and in relation to his or her goals, expectations, parameters and social relations. It is a wide-ranging concept affected in a complex way by the person’s physical health, psychological state, level of independence, social relationships and his or her relation to prominent features of the environment [5, 6]. On the other hand, and in order to incorporate the overall aspects of QoL that can affect health, the term health-related quality of life (HRQoL) has been introduced. HRQoL is the part of the quality of life concept that can be influenced by health and health care activities and excludes aspects of QoL that are not related to health like cultural, political or societal attributes [7].

Owing to the ongoing COVID-19 pandemic, it is a reality that the world has to live with the novel coronavirus, and hence, it is imperative to examine the QoL in the ongoing COVID-19 pandemic and to pay effort to understand QoL and the psychological and demographic factors that would indeed facilitate in the rehabilitation of people [8–10].

According to previous studies on epidemics and pandemics, the general population's QoL is impacted and influenced by a variety of demographic, social, and health-related factors. As a result, a more thorough assessment of the impact of the COVID-19 pandemic on QoL is required. Such information is critical for determining the COVID-19 burden in various geographical and cultural areas, as well as over the course of the pandemic. Hence, the aim of this study is to measure quality of life of the public throughout COVID-19 crisis and factors affecting it. This study represents one of the first surveys conducted on the general population in Dakahlia which may assist governmental agencies and healthcare professionals in deeper perception of factors affecting the QoL of the public in order to safeguard the highest possible quality during the battle against COVID-19.

## Methods

### Research design and setting

This is a community-based cross-sectional household study with an analytic component conducted in Bani Ebaid City for 7 months from August 2020 to March 2021. This city is an agricultural area in Dakahlia governorate located 27 km east of Mansoura City and the residential block in the city of Bani Ebaid is approximately 11 km^2^. The city is a recently urbanized rural community, where most people are agricultural workers. According to the Egyptian Central Agency for Public Mobilization and Statistics [11], the estimated total population of Bani Ebaid City was 42,235 persons, with males representing 51.2% of the city population.

### Study participants

The study targeted adult Egyptians between 18 and 80 years old, both males and females who are residing in Bani Ebaid and willing to participate in the study. Medical personnel (physicians and nurses) and persons with a history of psychiatric disorders were excluded from the study.

### Sample size

An external pilot study was conducted, and the estimated mean of the total QoL score was used in calculating the sample size. The external pilot study revealed a COVID-19 impact on QoL score mean ± standard deviation of 2.35 ± 0.6. Considering 5% alpha error, 20% beta error and 3% level of precision, sample size was calculated, using the MedCalc program for Windows (version 14.8.1), to be 498 individuals at least and the full-scale study included 500 individuals.

### Data collection

The city map was obtained and divided into 6 sectors and the main street in each sector was defined. The first house to the right in the main street of each sector was selected and then a systematic technique was adopted to include every 10^th^ house where only the first person who meets the investigator and fulfilling all inclusion criteria was allowed to participate in the study.

During the data collection process, strict infection control measures were implemented. All participants were given surgical masks and gloves, to wear during the interview. The researcher and participants washed their hands frequently with soap and water or alcohol-based hand rub when available. Furthermore, study participants were asked to stay 1.5 m away to maintain social distance, and windows were kept open when indoors to promote good ventilation.

Ethical approval for this study was acquired from Institution Research Board (IRB) (code number: MD.20.06.337). Informed consent was obtained from respondents and the collected data were not used for any other purpose.

### Measurements

Data were collected through structured interviews, and the collected data included socio-demographic characteristics including age, sex, employment, income, education, marital status, as well as some data related to smoking behavior and presence or absence of chronic disease (including hypertension, diabetes mellitus, cardiovascular disease, bronchial asthma, etc.). Also, their experience with COVID-19 as previous COVID-19 infection, know someone infected with COVID-19, know someone who deceased of COVID-19, and been ill for more than one day with symptoms including any of the following: fever, dry cough, difficulty of breathing, sore throat, loss of smell/taste, headache or diarrhea during 14 days previous to the interview.

Data about QoL were collected using the COV19-Impact on Quality of Life (COV19-QoL) scale. The COV19-QoL scale is a recently developed specific reliable and valid tool assessing perceptions of deterioration in QoL as a result of the COVID-19 pandemic. The phrase " Due to the spread of the coronavirus” was used to introduce the various items, to relate the change in QoL to the COVID-19 pandemic. Cronbach’s alpha coefficient of the original English version was 0.88 [12].

The COV19-QoL scale covers 6-items that are thought to be mainly affected during the outbreak. It includes questions about patients’ feelings about the impact of the current pandemic on their QoL in general, the possible mental and physical health deterioration among participants, the levels of anxiety and depression due to the pandemic and lastly, the extent to which participants perceive their personal safety is now in danger.

### Scale translation, validity and reliability

The COV19-QoL scale was translated into Arabic by two independent translators to reach the initial Arabic version of the questionnaire. Back translation of the Arabic version into English was done by two other translators in order to guarantee that the translation was appropriate and that the synonyms were consistent. The content validity indices (CVI) were considered by 10 expert jurors, who were academic professionals in the field, with regards to clarity, relevance, and translation, and the scale was judged as a valid tool [13]. The COV19-QoL scale Arabic version item CVI (I-CVI) for relevance and clarity was between 0.89 and 1. For both relevance and clarity, the scale CVI (S-CVI) was 0.945. In contrast, the expert CVI (E-CVI) ranged in relevance and clarity between 0.7 and 1.0.

The original questionnaire used a five-point Likert scale. However, based on the expert jurors, it was suggested that it would be easier and more understandable in the local culture to use a three-point Likert scale (agree, not sure, and disagree). Disagree is scored as 1, not sure is scored as 2, agree is scored as 3. The total score is calculated by averaging the sum of all items score and a higher score indicates a lower QoL.

A pilot study was performed, including 50 individuals from the 6 sectors of the city (8–9 participants from each sector) to ensure representativeness of the pilot study participants. Internal consistency of the final validated Arabic version was evaluated based on the pilot study results using Cronbach’s Alpha reliability coefficients. Results revealed acceptable Cronbach’s Alpha coefficients (Cronbach's Alpha = 0.795).

### Data analysis

Data were coded, processed and analyzed using IBM SPSS Statistics for Windows, Version 25.0 (IBM Corp, 2017). Categorical data were presented as numbers and percentages of the total, while continuous data were expressed as mean ± standard deviation. Student's t-test was used to compare normally distributed continuous data between 2 groups. Stepwise multiple linear regression analysis was applied to evaluate the contribution of factors found to be significant in bivariate analysis in predicting QOL and COVID-19 total score. Qualitative variables were included in the model as dummy variables. They are coded as 0: age < 40 years, male sex, not employed, sufficient income, > secondary education, not married, not smoker, no comorbidities, no previous COVID-19 infection, not know someone infected with COVID-19, not know someone died with COVID -19, no symptoms suggestive of COVID-19 in the past 14 days, and the second half of data collection period. 1 was given for age ≥ 40 years, female sex, employed, insufficient income, ≤ secondary education, married, smoker, presence of chronic diseases, previous COVID-19 infection, personally know someone infected with COVID-19, personally know someone died with COVID-19, presence of symptoms suggestive of COVID-19 in the past 14 days, first half of data collection period. P value ≤ 0.05 was considered statistically significant.

## Results

### General characteristics of the participants

A total of 500 respondents were included in the study, of which 60.6% were < 40 years and 54.4% were males. About half of the respondents were employed (55.6%), had sufficient income (53.8%) and had a high level of education (53.2%). 62.2% of the participants were married. The majority were nonsmokers (81.8%) and were free from chronic disease (79%) (Table 1).

**Table 1:** Characters of study population

Variables	Total N (%) N = 500
Age	
< 40 years	303 (60.6)
≥ 40 years	197 (39.4)
Sex	
Male	272 (54.4)
Female	228 (45.6)
Employment	
Employed	278 (55.6)
Not employed/retired	222 (44.4)
Monthly income	
Sufficient	269 (53.8)
Insufficient	231 (46.2)
Educational level	
Basic	98 (19.6)
Secondary	136 (27.2)
Higher	266 (53.2)
Marital status	
Married	311 (62.2)
Not married	189 (37.8)
Smoking	
Smoker	91 (18.2)
Not smoker	409 (81.8)
Chronic diseases	
Absent	395 (79)
Present	105 (21)
Previous Covid-19 infection	128 (25.6)
Personally know someone infected with COVID-19	350 (70)
Personally know someone who died of COVID-19	178 (35.6)
Having a symptom suggestive of COVID-19 in past 14 days	123 (24.6)

### QoL score

The total COV19-QoL scale score (mean ± standard deviation) was 2.3 ± 0.6. Two items show the highest mean with 2.6 ± 0.7 (quality of life in general and perception of danger on their personal safety) indicating the poorest quality of life regarding these 2 items. However, the lowest mean score is related to the perception of mental health deterioration (1.9 ± 0.8) (Table 2).

**Table 2:** Distribution of participants’ responses to quality of life questions

Due to the spread of the coronavirus	N (%)			Mean ± SD
	Disagree	Not sure	Agree	
I think my quality of life is lower than before	61 (12.2)	59 (11.8)	380 (76)	2.6 ± 0.7
I think my mental health has deteriorated	216 (43.2)	117 (23.4)	167 (33.4)	1.9 ± 0.8
I think my physical health may deteriorate	149 (29.8)	103 (20.6)	248 (49.6)	2.1 ± 0.9
I feel more tense than before	87 (17.4)	72 (14.4)	341 (68.2)	2.5 ± 0.8
I feel more depressed than before	149 (29.8)	100 (20)	251 (50.2)	2.2 ± 0.87
I feel that my personal safety is at risk	67 (13.4)	49 (9.8)	384 (76.8)	2.6 ± 0.7
COV19-QoL (total scale)				2.3 ± 0.6

SD: standard deviation, COV19-QoL: COV19-Impact on Quality of Life

### QoL score and participants characteristics

A statistically significant higher mean COV19-QoL scale score (indicating poor quality of life) was observed in people aged 40 years and older compared to those less than 40 years (mean ± standard deviation, 2.5 ± 0.5 versus 2.27 ± 0.6, p value < 0.001). Also, females had higher mean COV19-QoL scale score than males (2.43 ± 0.5 versus 2.27 ± 0.6, p value = 0.001). Mean COV19-QoL scale score was also found to be significantly higher in participants with insufficient monthly income compared to sufficient (2.42 ± 0.5 versus 2.28 ± 0.6, p value = 0.01), ≤ secondary compared to > secondary educational level (2.41 ± 0.5 versus 2.3 ± 0.6, p value = 0.03), married compared to unmarried (2.42 ± 0.5 versus 2.2 ± 0.6, p value < 0.001) and participants with comorbidities compared to absent comorbidities (2.5 ± 0.6 versus 2.3 ± 0.6, p value = 0.004) (Table 3).

**Table 3:** Distribution of QoL during COVID-19 pandemic according to sociodemographic variables and COVID-19 experience

Variables	COV19-QoL score	t-test	P value
	Mean ± SD		
Age			
< 40 years	2.27 ± 0.6	3.9	≤ 0.001
≥ 40 years	2.5 ± 0.5		
Sex			
Male	2.27 ± 0.6	3.2	≤ 0.001
Female	2.43 ± 0.5		
Employment			
Employed	2.33 ± 0.6	0.46	0.6
Not employed/retired	2.36 ± 0.6		
Monthly income			
Sufficient	2.28 ± 0.6	2.55	0.01
Insufficient	2.42 ± 0.5		
Educational level			
≤ secondary	2.41 ± 0.5	− 2.2	0.03
> secondary	2.3 ± 0.6		
Marital status			
Married	2.42 ± 0.5	3.8	≤ 0.001
Not married	2.2 ± 0.6		
Smoking		0.46	0.6
Smoker	2.35 ± 0.6		
Not smoker	2.32 ± 0.6		
Chronic diseases			
Absent	2.3 ± 0.6	2.9	0.004
Present	2.5 ± 0.6		
Previous Covid-19 infection			
No	2.348 ± 0.56	0.053	0.9
Yes	2.345 ± 0.55		
Personally know someone infected with COVID-19			
No	2.19 ± 0.6	4.129	≤ 0.001
Yes	2.4 ± 0.5		
Personally know someone who died of COVID-19			
No	2.3 ± 0.6	2.1	0.04
Yes	2.42 ± 0.6		
Have a symptom suggestive of COVID-19 in past 14 days			
No	2.32 ± 0.6	1.7	0.09
Yes	2.42 ± 0.6		
Duration			
First half of data collection period	2.45 ± 0.6	− 3.9	< 0.001
Second half of data collection period	2.26 ± 0.6		

SD: Standard deviation, COV19-QoL: COV19 – Impact on Quality of Life, t-test: Student's t-test

Compared to their corresponding groups (Table 3), respondents who knew a person infected with COVID-19 (2.4 ± 0.5 versus 2.19 ± 0.6, p value < 0.001), those who know someone died of COVID-19 (2.42 ± 0.6 versus 2.3 ± 0.6, p value = 0.04), and study participants who shared in the first half of data collection period (2.45 ± 0.6 versus 2.26 ± 0.6, p value < 0.001) were more likely to have higher mean COV19-QoL scale score, indicating lower quality of life.

### Predictors of QoL score

Significant variables in the bivariable analysis were entered into multiple linear regression analysis model, which revealed that sex (regression coefficient (95% CI) = 0.1 (0.02 to 0.2), p value = 0.02), monthly income (regression coefficient (95% CI) = 0.1 (0.004 to 0.2), p value = 0.04), knowing someone infected with COVID-19 (regression coefficient (95% CI) = 0.15 (0.08 to 0.3), p value = 0.001), and data collection time (regression coefficient (95% CI) = 0.1 (0.006 to 0.2), p value = 0.04) were the independent predictors for overall QoL scale score (Table 4).

**Table 4:** Multiple linear regression of significant independent predictors of COVID-19-Impact on Quality of Life

Variables	Bivariate linear regression		Multiple linear regression model	
	β (95% CI)	P value	β (95% CI)	P value
Age	0.17 (0.1 to 0.3)	< 0.001	0.09 (− 0.009 to 0.2)	0.07
Sex	0.14 (0.06 to 0.26)	0.001	0.1 (0.02 to 0.2)	0.02
Employment	− 0.02 (− 0.1 to 0.08)	0.6		
Monthly income	0.1 (0.03 to 0.2)	0.01	0.1 (0.004 to 0.2)	0.04
Educational level	0.09 (0.01 to 0.2)	0.03		
Marital status	0.17 (0.1 to 0.3)	< 0.001		
Smoking	− 0.02 (− 0.16 to 0.1)	0.6		
Comorbidities	0.13 (0.06 to 0.3)	0.004		
Previous Covid-19 infection	− 0.002 (− 0.1 to 0.1)	0.9		
Personally know someone infected with COVID-19	0.18 (0.12 to 0.3)	< 0.001	0.15 (0.08 to 0.3)	0.001
Personally know someone who died of COVID-19	0.1 (0.008 to 0.2)	0.04		
Have a symptom suggestive of COVID-19 in past 14 days	0.07 (− 0.02 to 0.2)	0.1		
Duration	0.17 (0.1 to 0.3)	< 0.001	0.1 (0.006 to 0.2)	0.04
			Constant	2.03
			Model F	9.1
			Model R^2^	0.1
			P value	< 0.001

β: regression coefficient, CI: Confidence Interval, Model F: Model Analysis of Variance F test, Model R^2^: Model R square. Qualitative variables were included in the model as dummy variables. They are coded as 0: age < 40 years, male sex, not employed, sufficient income, > secondary education, not married, not smoker, no comorbidities, no previous COVID-19 infection, not know someone infected with COVID -19, not know someone died with COVID -19, no symptoms suggestive of COVID-19 in the past 14 days, the second half of data collection period

## Discussion

The speedy transmission of the novel coronavirus along with instant measures in response to the COVID-19 pandemic has led to multiple encounters that imposed deviations from the performance related to healthy lifestyles of people of different ages and consequently adversely affecting QoL [14]. In this study, the COV19-QoL scale mean ± standard deviation is 2.3 ± 0.6. However, this score is lower compared to the QoL of Iranian individuals (mean = 2.73) [15], and Filipino teachers (mean = 2.75) [16] and much lower than the score of people free from mental health problems in Croatia (mean = 2.91) [12]. Although this indicates less adverse effect on the QoL of study participants compared to others, the results of this work still suggest that the international spread of COVID-19 has definitely impacted the lives of the public.

Moreover, this study finds that the biggest influence of the pandemic is on personal safety and the QoL in general whereas the mental aspect was minimally altered. This is similar to previous studies [12, 16, 17]. Low impact on mental health could be based on the fact that COVID-19 is a physical health threat and usually the public from developing countries is not aware of their mental health and more focused on the physical aspect of health only especially during the epidemic [18].

This study finds that some individuals are more prone to poor QoL than others during the pandemic due to some socio-demographic background, financial status and experience with COVID-19 as verified by the multiple regression model. For instance, COVID-19 impacted the QoL more significantly in females than males. This is in agreement with studies where higher stress levels were reported from women than their men colleagues among students in a Turkish nursing school [19], nursing students in the Philippines [20], teachers and students in the Philippines and Chile [21–23] and among the public in Australia [24], Italy [25], and Brazil [26]. This can be explained by the fact that women in these countries play a central role in all aspects related to the family. Moreover, hormones and overthinking about social situations make women more emotional and stressed [17]. In contrast, male participants were more at risk of lower QoL in research from the Kingdom of Saudi Arabia [27]. This observation can be explained by the fact that in KSA male individuals are the main family supporter and were likely more stressed out of fear about their health or unemployment during the pandemic. Furthermore, the lockdown restrictions deprived men of their routine social and religious activities [27]. All these factors impacted males QoL more than females.

Although an important factor to study, limited number of related studies endorsed the effect of income on QoL during COVID-19 [28]. However, a study [15] that included it did not find a significant difference between different income groups in contrast to this study. However, prior research involving income and QoL relationship showed that low income is related to poor QoL [29, 30] Increased public concern about financial conditions and economic pressure could exacerbate the pandemic influence on QoL. Individuals are put in an unpleasant situation due to financial insecurity, which has an impact on their QoL and overall well-being [31].

However, the QoL was affected the most significantly when a relative was diagnosed with COVID-19. This is similar to the reported finding from Brazilian studies [26, 32]. Suffering the disease by self or by a close family relative leads to anxiety of health complications and outcome of the disease, thus impacting the perceived QoL [21, 33, 34]. This is unsurprising considering the isolation of loved ones, difficulty in their caring, the inability of a family member to provide support, and prolonged recovery time. Such stressors have been linked to survivors' family members' poor QoL and psychological health [35].

The findings demonstrated that the initial half of the data collection time yielded lower quality of life than the second half. Therefore, it could be suggested that people became increasingly resistant to the pandemic situation and restriction changes over time, possibly because they had coped with the pandemic-related events and got accustomed to the uncertainty and frequent policy changes [36]. Similarly, recent findings reported poor QoL in earlier waves of the pandemic compared to late pandemic, indicating that adapting to changes in daily life has reduced the anticipated impact on Qol over time [37]. However, previous studies suggested that the QoL of populations during the COVID-19 pandemic does not differ over time [38]. Further research is needed to confirm the current and earlier findings.

This study also detected some significant factors associated with QoL. For example, people aged 40 years and more presented lower QoL and this was in contrast with a Brazilian study which presented individuals aged ≥ 40 years with better QoL [26]. On the other hand, Rabacal et al., 2020 in their study on Filipino teachers failed to detect any significant difference in the QoL in relation to age [16]. Also, the current study emphasizes that people with lower levels of education have poor QoL, which is consistent with prior findings [8]. People with lower levels of education do not have access to appropriate healthcare and have a lower level of resilience during the COVID-19 pandemic than people with a higher level of education [39]. Education experiences alter one's life's purpose and are linked to happiness and satisfaction and have a significant impact on income [26]. However, this finding doesn’t support the previous work which showed that poor QoL was observed in patients with a higher level of education, a greater level of awareness and concern of COVID-19 and its impact on life [40]. Others revealed no association between QoL and educational level [41].

In the present study, married participants reported poorer QoL compared to singles. However, the relation between QoL and marriage is not conclusive as some studies reported the opposite [15, 26] and another one could not detect any significant difference in the QoL relative to marital status [16]. The inconsistency in determining the role of sociodemographic like age, gender and marital status is more or less based on the social context which determines the role played and the amount of social, financial and emotional pressure expressed on different classes in different societies [42]. This study findings revealed that respondents with co-morbidities have significantly lower QoL scores. This result is consistent with the findings of previous studies [27, 43–45]. This can be explained by the fact that patients with chronic comorbidities need either critical or continuous medical attention which was limited during lockdown imposing more stress and worsening their diseases conditions [30].

Some limitations of this study should be considered. The study only involved participants from a single locality, so the study findings cannot be generalized to all Egyptians. These findings need to be confirmed by other studies involving a bigger sample that may also include a qualitative element. Also, this is a one-time cross-sectional study that could not capture the ongoing effects of the COVID-19 pandemic on various dimensions of QoL. Moreover, the lack of knowledge about QoL before covid-19 to compare with hinders the value of this study in assessment of the actual effect of COVID 19 on QoL.

## Conclusions

COVID-19 pandemic has a persuasive effect on the quality of life of the Egyptian public. More attention should be directed to people with significant predictors that increase the vulnerability to poorer quality of life like female sex, insufficient monthly income and persons knowing someone infected with COVID-19. Finally, further research with longitudinal design and a qualitative component is highly recommended to detect and deeply understand the changes in QoL also involving a larger population is important to generalize the findings.

## Data Availability

The datasets used and/or analyzed during the current study are available from the corresponding author on reasonable request.

## Abbreviations

QoL: Quality of life
COVID-19: Coronavirus disease 2019
HRQoL: Health-related quality of life
COV19-QoL: COV19-Impact on Quality of Life
CFR: Case fatality ratio
CVI: Content validity index
I-CVI: Item-Content validity index
S-CVI: Scale-Content validity index
E-CVI: Expert-Content validity index
